# Drivers of the range expansion of the European catfish (*Silurus glanis*) within its native distribution

**DOI:** 10.1111/jfb.70099

**Published:** 2025-06-12

**Authors:** Jan Baer, Stephan Hüsgen, Matthias Fromherz, Juergen Geist, Alexander Brinker

**Affiliations:** ^1^ Fisheries Research Station of Baden‐Wuerttemberg Langenargen Germany; ^2^ Regierungspräsidium Karlsruhe Fisheries Administration Karlsruhe Germany; ^3^ Aquatic Systems Biology Unit Technical University of Munich Freising Germany; ^4^ University of Konstanz Constance Germany

**Keywords:** apex predator, fisheries management, gobiids, non‐native species, stomach content analysis

## Abstract

The European catfish *Silurus glanis* L. continues to spread as an invasive species in Europe. Meanwhile, increasing abundance and range expansions are also suspected within its native distribution. The objective of this study was to characterize the feeding, growth and abundance of *S. glanis* in 12 lakes and 12 rivers within its European native range in relation to environmental changes that may explain population trends over time. The results suggest a shift in factors limiting the carrying capacity of catfish habitats within this range, leading to increases in growth and abundance. Statistical modelling identified warming and increasing population density of invasive gobies as likely contributors to this shift. Given that catfish predation on native species can be expected to increase dramatically, there is an urgent need for new management strategies. It is recommended that catfish removal rates should be increased, especially in areas of high importance for native fish and crayfish species, such as spawning grounds and at bottlenecks for migration.

## 1 INTRODUCTION

Large apex predators are rare in nature and among fish, as with other taxa, populations of many such species are in decline (He et al., 2019). In contrast, stocks of European catfish *Silurus glanis* L. (hereafter, catfish), a freshwater apex predator, are increasing outside the species' native range (Cucherousset et al., 2018). Individuals of this species are able to reach over 2.7 m in length and achieve body weights of over 130 kg (Boulêtreau & Santoul, 2016), making it one of the 20 largest freshwater fishes in the world (Stone, 2007). Its native range includes the Baltic, Black, Caspian and Aral Sea basins of Eastern Europe, with the most westerly extent being the southern part of the River Rhine drainage in Germany (Kottelat & Freyhof, 2007). Due to their popularity with anglers and for different biomanipulation projects (Vejřík, Vejříková, Blabolil, et al., 2017), catfish have been introduced into many areas outside their natural distribution range and the species is now also frequently found in freshwater systems outside Eurasia (Claudia & Doina, 2013; Cunico & Vitule, 2014; Schlumberger et al., 2001) as well as in western and southern Europe (Boulêtreau et al., 2021; Carol et al., 2007; Vejřík, Vejříková, Blabolil, et al., 2017). New management strategies to prevent further spreading and future introductions in southern European lakes and reservoirs are on the way (De Santis et al., 2024). Meanwhile, new scientific evidence suggests stocks of catfish are also increasing within the native range (Fromherz et al., 2024) where the factors contributing to population changes are hardly studied. Given that food web‐related bottom‐up control mechanisms generally keep the abundance of apex predators within an established range (hereafter: carrying capacity, given as number of individuals in an area; Chapman & Byron, 2018) consistently rather low (Haugen et al., 2007; Hayward et al., 2007; McQueen et al., 1989; Minns et al., 1996), this may point to changes in habitat conditions and associated food web structures change over time. However, a declining trend in the abundance of native prey fish species in Europe (Benitez et al., 2022; Gozlan et al., 2019; Mueller et al., 2018) might be expected to reduce carrying capacity for apex predators such as catfish. Thus, the newly recorded increases within its native range are a surprising result requiring validation and targeted study to understand the drivers of the expansion and its potential ecological consequences. Further investigation is rendered particularly urgent by the well‐known dramatic impacts resulting from high catfish densities elsewhere, for example in their non‐native range on the Iberian Peninsula where in the absence of other piscivorous fish, native small‐bodied fish have been driven to the brink of extinction by catfish predation (Copp et al., 2009; De Santis et al., 2024; Ferreira et al., 2019).

Alongside the wealth of research addressing the ecological effects of catfish introduction (Carol et al., 2009; Ferreira et al., 2019; Vagnon, Bazin, et al., 2022; Vejřík et al., 2019), there is, to the best of our knowledge, only one study in natural waters within the species' native range where a high density of *S. glanis* negatively affected a local food web, which was additionally impacted by anthropogenic stressors (Wysujack & Mehner, 2005). Further empirical investigation of the consequences of increasing abundance within native catfish areas is therefore urgently needed, especially since recent modelling predicts significant range expansion due to climate change (Basen et al., 2022). If this development and concomitant ecosystem impacts are confirmed, new approaches will be urgently required in fisheries management to deal with this apex predator (Vejřík et al., 2024).

**FIGURE 1:**
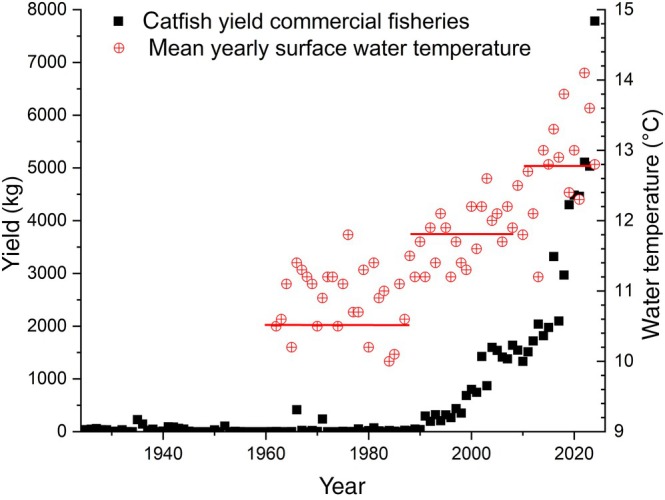
Annual commercial yields of catfish (*Silurus glanis*) in Upper Lake Constance from 1924 to 2024 (black squares) and yearly mean water temperatures (red circles with cross), measured at 0.5 m depth between 1962 and 2024. The three horizontal red lines represent mean surface water temperatures for the periods 1962 to 1989 (10.9°C), 1990 to 2009 (11.8°C) and 2010 to 2023 (12.8°C).

In the federal state of Baden‐Württemberg, southern Germany, *S. glanis* was a very rare endemic species found only occasionally in the Rivers Danube and Rhine, and in Lake Constance until the end of the 13th century (Dußling et al., 2018). During the 20th century however, the abundance of this species began increasing state‐wide, and the trend has gathered pace over the last 20 years (Dußling et al., 2018). In Upper Lake Constance between 1924 and the end of the 1980s, the species was caught in very small quantities, not normally exceeding 50 kg per year (Figure 1). Even with no stocking, stable fishing pressure and consistent fishing practice (DeWeber et al., 2022), yields began to increase continuously from the beginning of the 1990s to current catches of above 3000 kg a year and a new record in 2024 with 7800 kg (Figure 1). This unprecedented development is surprising, given that the densities of important prey species like perch (*Perca fluviatilis* L.) (Čech et al., 2012; Vejřík, Vejříková, Kočvara, et al., 2017) or whitefish (*Coregonus* spp.) (Vagnon, Cattanéo, et al., 2022) in this and other pre‐alpine lakes have been declining steadily for decades (Baer, Eckmann, et al., 2017; Vagnon, Bazin, et al., 2022). However, the increase coincidences with strong warming of the lake (Figure 1). Catfish require several weeks of water temperatures of at least 22–23°C for spawning, and larvae die at temperatures below 13–14°C (Copp et al., 2009). Thus, the warming of Lake Constance might have benefited catfish reproduction and recruitment, permitting stock development of this formerly rare species. However, further data is necessary to confirm temperature as a driver of species expansion.

Meanwhile, the densities of potentially relevant new prey in the catfish's native range, i.e. non‐native fish and crayfish (Copp et al., 2009), have been increasing (Baer, Hartmann, & Brinker, 2017; Chucholl, 2016; Mikl et al., 2017), and anglers and commercial fishers report large number of these taxa in the stomachs of caught catfish. The positive effect of non‐native prey on native predator populations can be extremely pronounced (Pintor & Byers, 2015), but no studies have been conducted to validate this.

Additionally, many small lakes and gravel pits in southern Germany now also contain catfish, most likely as a result of widespread stocking. The impact on local fish communities is largely unknown, but given the variety of well‐known potential ecological consequences, more data about potential drivers of catfish abundance is urgently needed. Furthermore, data on feeding patterns and growth rates needed to inform models to assess the local influence of native catfish on fish fauna and to define the species' ecological niche are largely missing. Only with such data at hand can informed decisions be taken regarding potential management actions, such as stricter rules for fishing and the removal of the catch (Fromherz et al., 2024).

The principle of bottom‐up control of apex predators (Wallach et al., 2015) in environments with stable habitat availability (Haugen et al., 2007) suggests that populations of catfish in their natural range were previously limited by the availability of native prey species. However, based on observations in Lake Constance, we hypothesize that local conditions which historically limited the carrying capacity for catfish have changed. Factors such as ongoing climate change or the presence of non‐native species and their interaction are suspected to positively affect catfish abundance. To study these hypotheses and to compile actual data on feeding patterns and growth from catfish, we conducted a state‐wide study in Baden‐Württemberg, southern Germany, sampling 12 rivers and 12 lakes in order to (i) develop time series to describe the development of catfish stocks within the species' native habitat range, (ii) identify and characterize potential drivers for growth and range expansion, and (iii) characterize the prey preferences of catfish to assess potential impacts on other species.

## 2 MATERIALS AND METHODS

All fish were caught by licenced personnel by permission of the local fisheries administration (Regierungspräsidium Karlsruhe, Freiburg, Stuttgart, Tübingen) and according to German animal protection legislation (§ 4) and the ordinance on the slaughter and killing of animals (Tierschutzschlachtverordnung § 13). All catfish sampled were euthanized with an overdose of clove oil (1 mL L^−1^) and a gill cut.

### 2.1 Study area

**FIGURE 2:**
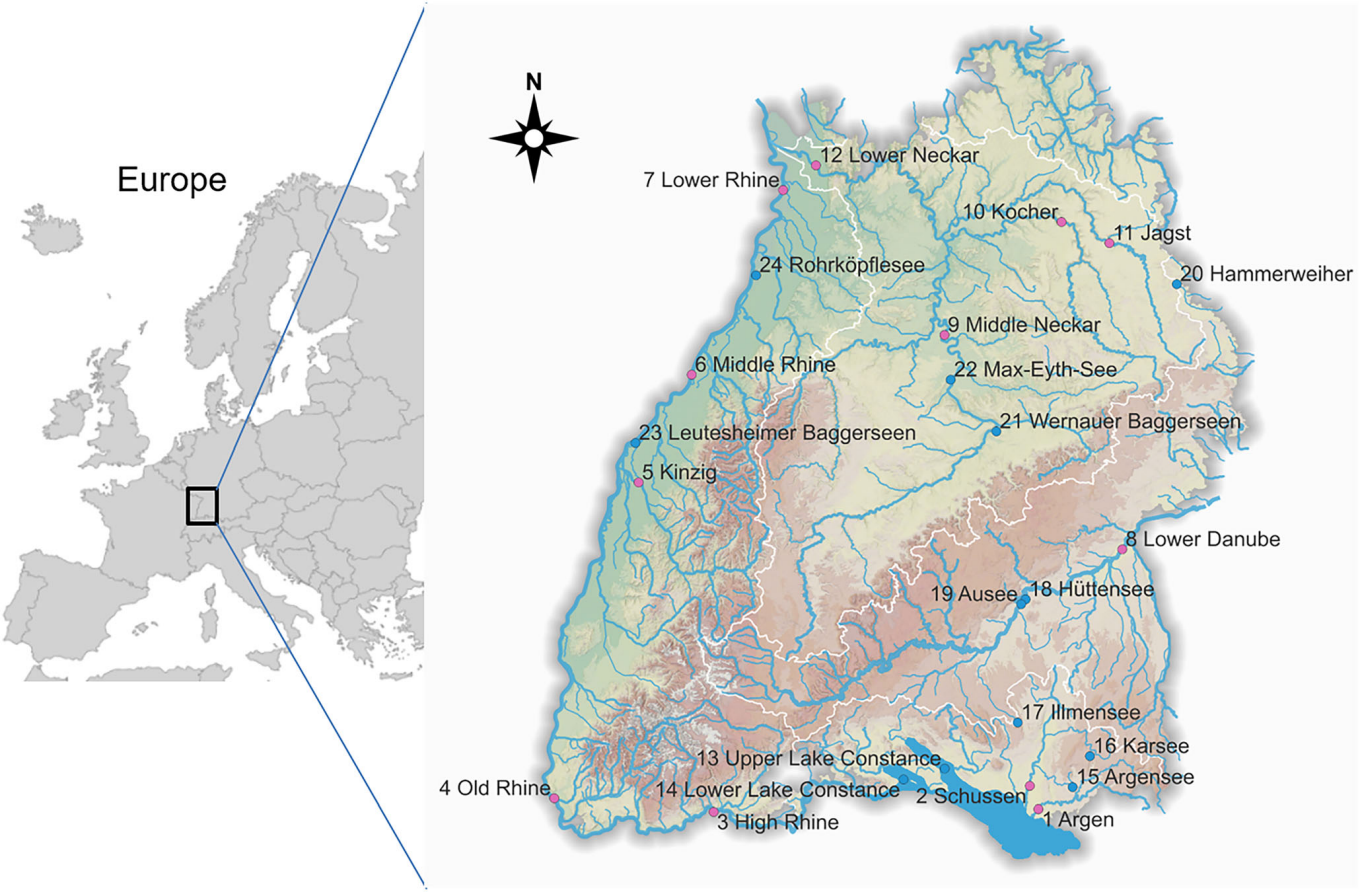
Map of study sites. Further information for every sampled river (red circles, 1–12) and every lake (blue circles, 13–24) is provided in Table 1.

The study was carried out in the federal state of Baden‐Württemberg, southwestern Germany (Figure 2). Four large catchments can be found here: (i) Lake Constance and its tributaries, (ii) the River Rhine flowing north on the borders with Switzerland and France, (iii) the River Neckar, from the middle of the state to its confluence with the Rhine in the north, and (iv) the River Danube, flowing east to the Black Sea. In these four large catchments, including their tributaries and nearby standing water bodies, catfish were sampled from 12 river stretches and 12 lakes and gravel pits (Figure 2). Only waters that appeared generally suitable for catfish were considered (slow‐moving lotic or lentic waters of considerable depth, occurrence of submerse macrophytes, warm during summer; Cucherousset et al., 2018). The altitude of each sampling site is given in the Supplement (Table 1). From each water body, we sampled at least 10 specimens (Table 1). In total, we sampled 573 individuals measuring between 6.1 and 235.4 cm (mean total length ± standard deviation [SD] = 58.1 ± 41.0 cm).

**TABLE 1:** List of sampled rivers and lakes, their width (rivers) or surface areas (lakes), number of sampled catfish per water body and types of sample taken in each river and lake (for more details see text).

Study site (number on map)	Catch‐ment	Mean width (m, river) or surface (ha, lake)	Samp‐led cat‐fish (n)	Samp‐ling for growth rates	Samp‐ling to compare growth at age class 2 and 3	Samp‐ling for growth during differed time periods	Samp‐ling for develop‐ment of abun‐dance	Samp‐ling for diet
Rivers								
Argen (1)	Rhine	15	14	x	x			x
Schussen (2)	Rhine	15	27	x	x			x
High‐Rhine (3)	Rhine	60	29	x				x
Old Rhine (4)	Rhine	80	37	x	x			x
Kinzig (5)	Rhine	25	26	x	x			x
Middle Rhine (6)	Rhine	120	29					x
Lower Rhine (7)	Rhine	200	27	x	x	x	x	x
Lower Danube (8)	Danube	30	18	x				x
Middle Neckar (9)	Neckar	40	12					x
Kocher (10)	Neckar	25	10	x				x
Jagst (11)	Neckar	25	51	x	x			x
Lower Neckar (12)	Neckar	100	35	x	x		x	x
Lakes								
Upper Lake Constance (13)	Rhine	47,200	113	x	x			x
Lower Lake Constance (14)	Rhine	6200	15	x		x	x	x
Argensee (15)	Rhine	26	11	x	x			x
Karsee (16)	Rhine	3	11	x				x
Ilmensee (17)	Danube	64	25	x	x			x
Hüttensee (18)	Danube	5	16	x				x
Ausee (19)	Danube	5	13	x				x
Hammer‐weiher(20)	Danube	5	10	x				x
Wernauer Baggerseen (21)	Neckar	20	13	x				x
Max‐Eyth‐See (22)	Neckar	17	10	x				x
Leutesheimer Baggersee (23)	Rhine	22	11	x				x
Rohrköpflesee (24)	Rhine	58	10	x	x			x

### 2.2 Sampling

Sampling was conducted between April 2022 and November 2024 (Table S1). Most waters were fished at least twice and sampling took place in all months except December and February. Most catfish were captured by electrofishing (EFKO, straight DC, 300–600 V, 8 kW), in most instances from a boat, but in some smaller streams by wading. In Lower Lake Constance, all catfish were caught by gillnets during a scientific fishing campaign (September 2024). In Upper Lake Constance, all catfish exceeding 1 m total body length were caught in a commercial fyke net (6 mm mesh size) set in April and May 2022 and 2023. In three small lakes (Hammerweiher, Max‐Eyth‐See and Rohrköpflesee) and one river (River Jagst) sampling was carried out exclusively by anglers, who stored the catfish whole in a freezer (−18°C) and transported them later to the Fisheries Research Station for further analysis. Wet weight (to the nearest g) and total length (TL, to the nearest cm) were recorded for all catfish shortly after capture or shortly after defrosting.

### 2.3 Growth

The first two to four vertebrae were dissected from 10 randomly selected catfish from all 12 lakes and from 10 river sections. The aim was to achieve almost equipartition of individual size classes, therefore if certain length classes were missing or overrepresented in these 10 animals, additional individuals were included (Table 1). Vertebrae were boiled in water, cleaned from remaining tissue, dried and photographed. Cracked or damaged vertebrae were excluded from further analysis. Images of intact vertebrae (at least two per individual) were then analysed with a computer and growth band pairs counted (Alp et al., 2011). To compare length at age from the sampled sites, a von Bertalanffy growth function (VBGF) was applied:

(1) $$L_{t}=L_{\infty }\left[1-e^{-k\left(t-t_{0}\right)}\right]$$

where *L*_*t*_ is the length at age *t*, $L_{\infty }$ is the asymptotic length, *k* is the growth coefficient, *t* is the age (year) and *t*_0_ is a constant.

Potential impacts of certain variables on growth of *S. glanis* according to the VBGF were investigated using the following general linear model (GLM) (Sachs, 1997):

(2) $$Y_{\text{ijklm}}=\mu +\alpha_{i}+\beta_{j}+\delta_{k}+\varepsilon_{l}+\zeta_{m}+\eta_{\text{ijklm}}$$

where *Y*_*ijklm*_ is length at age class 10 in cm, *μ* is overall mean, *α*_*i*_ denotes the scale of the sampled lakes or rivers (small: rivers <20 m width and lakes <50 ha; medium: rivers with a width of 20–40 m and for lakes of 50–100 ha; large: rivers >40 m width and lakes >100 ha in area; Table 1), *β*_*j*_ is the mean number of days of summer conditions (days with ≥25°C air temperature) in the 10 years prior to sampling (DWD 2024) as a proxy for heat budget, *δ*_*k*_ is the occurrence of gobiids (yes or no) at the sampling site [here: round goby *Neogobius melanostomus* (Pallas 1814), tubenose goby *Proterorhinus semilunaris* (Heckel 1837), or bighead goby *Ponticola kessleri* (Günter 1861), *ε*_*l*_ is the occurrence of non‐native crayfish (yes or no) at the sample site [here: *Faxonius immunis* (Hagen 1870), *Faxonius limosus* (Rafinsque 1817), *Procambarus clakii* (Girard 1852), *Pacifastacus leniusculus* (Dana 1852)], *ζ*_*m*_ is the type of sampled site (lake/river), and *η*_*ijklm*_ is random residual error.

### 2.4 Growth at age 2 and 3

For a back‐calculation of length at age 2 and 3 years (when growth rates are highest; Copp et al., 2009), we used images of 457 intact vertebrae from a total of 194 catfish (between two and three vertebrae per individual and between six and 49 per site) from seven rivers and four lakes (Table 2). According to the Fraser‐Lee method (Ricker, 1992) the total length of the fish (*L*_*i*_) at age *i* was estimated as:

(3) $$L_{i}=\frac{{\mathrm{VR}}_{i}}{{\mathrm{VR}}_{c}}\;\left(L_{c}-\alpha \right)+\alpha$$

where *L*_c_ is the total length of the fish at capture, *VR*_c_ is vertebral radius at capture, *VR*_*i*_ is vertebral radius at ring *i*, and *α* is the intercept of the regression. For all vertebrae, we measured *VR*_*i*_ up to an age of 3. The influence of air temperature on growth at age 2 and 3 between different years was tested with a general linear model using restricted maximum‐likelihood estimation (REML; Patterson & Thompson, 1971) as follows:

(4) $$\gamma_{\text{ijkl}}=\mu +\left(\alpha_{i}+\beta_{j}+\delta_{k}\right)+{\left(\eta \right)}_{l}+\varepsilon_{\text{ijkl}}$$

where *Y*_*ijkl*_ is yearly growth for age class 2 (*L*_2_ – *L*_1_) or age class 3 (*L*_3_ − *L*_2_), μ is the overall mean, *α*_*i*_ denotes sampling site (river or lake), *β*_*j*_ is the catchment (Table 1), *δ*_*k*_ is mean air temperature in the sampling area (DWD 2024) for the back‐calculated year, (*η*)_*l*_ is a random effect of the sampled river/lake and *ε*_*ijkl*_ is the random residual error.

**TABLE 2:** Total length (TL) of catfish in descending order at age class 10 according to the VBGF by sampling site and the influences of model terms.

Study site	TL (cm) at age 10 (VBGF)	Study site size category	Occurence gobiids	Occurence non‐native crayfish	Summer days per year	Type of water
Lower Neckar	135.8	Large	Yes	Yes	74	River
Lower Rhine	135.6	Large	Yes	Yes	76	River
Old Rhine	132.6	Large	Yes	Yes	78	River
Kinzig	128.5	Medium	Yes	Yes	78	River
Rohrköpflesee	128.2	Medium	No	Yes	77	Lake
Kocher	118.8	Medium	No	Yes	65	River
Jagst	117.2	Medium	No	Yes	58	River
Lower Danube	115.7	Medium	No	No	51	River
Upper Lake Constance	115.4	Large	No	Yes	67	Lake
Leutesheimer Baggersee	112.4	Small	No	Yes	78	Lake
Lower Lake Constance	110.8	Large	No	Yes	67	Lake
Hammerweiher	109.6	Small	No	No	53	Lake
Ilmensee	102.3	Medium	No	Yes	48	Lake
Hüttensee	101.0	Small	No	No	51	Lake
High‐Rhine	100.1	Large	No	Yes	70	River
Max‐Eyth‐See	99.2	Small	Yes	Yes	68	Lake
Wernauer Baggerseen	96.6	Small	No	Yes	69	Lake
Ausee	94.8	Small	No	Yes	52	Lake
Schussen	90.8	Small	No	Yes	61	River
Argensee	90.2	Small	No	No	55	Lake
Argen	89.5	Small	No	Yes	60	River
Karsee	86.5	Small	No	No	47	Lake
Model effects^a^		xx	x	n.s.	n.s.	n.s.

Abbreviations: TL, total length; VBGF, von Bertalanlanffy growth function.

^a^ xx = *p* < 0.01; x = *p* < 0.05; *n.s*., not significant.

### 2.5 Growth during different time periods

Growth in Upper Lake Constance and the Lower River Rhine was compared for catfish hatched in 2017, in 2012 and at the beginning of the 21st century (2005 for fish from Upper Lake Constance; 2001 for the Lower River Rhine). Growth was back‐calculated for the first 4 years of life (fish hatched in 2017), for the first 8 years of life (fish hatched in 2012) and for the first 12 years (older fish). Lengths at age 4 of all three groups and at age 8 for the 2012 and older cohorts were compared separately for both water bodies using a Kolmogorov–Smirnov test (Sachs, 1997). To express possible changes in air temperature at the different times and to use this data as a proxy for differing heat budgets, we calculated cumulative daily mean air temperature for both sites (DWD 2024) for the first 4 and 8 years of life of the sampled catfish.

### 2.6 Development of abundance

First, 20,000 sets of electric fishing data held in the database of the Fisheries Research station in Baden‐Württemberg were searched for data sets that could be used to reconstruct trends in catfish abundance in southern Germany over the last two decades. Only methodically coherent datasets were selected, i.e. those pertaining to stretches sampled by boat with an 8‐kW electrofishing unit at least annually from 2002 to 2023. For two rivers, the Lower River Rhine and the Lower River Neckar, several interconnected sections were identified along 25 km of river. For these, the catch per unit effort (CPUE) of catfish was calculated and standardized for a 100‐m stretch (shoreline length) and used as a proxy for abundance. To test for the possible influence of gobiids, known to be potentially key prey for catfish (Mikl et al., 2017), data sets were constructed using the same method as for catfish.

The influence of gobiid abundance (CPUE of gobiids) and air temperature on catfish abundance (CPUE of catfish) was tested with the following general linear model:

(5) $$\gamma_{\mathrm{ijk}}=\mu +\alpha_{i}+\beta_{j}+\left(\alpha_{i}*\beta_{j}\right)+\varepsilon_{\mathrm{ijk}}$$

where *Y*_*ijk*_ is catfish abundance (CPUE per 100 m), *μ* is the overall mean, *α*_*i*_ denotes the abundance of gobiids (CPUE per 100 m), *β*_*j*_ is the mean air temperature in the sampling year (°C) (DWD 2024), *α*_i_*β_*j*_ is the interaction between CPUE of gobiids and air temperature, and *ε*_*ijk*_ is the random residual error.

### 2.7 Stomach content

From all sampled catfish (Table 1) the stomachs were excised via an abdominal incision and separated from other visceral organs, opened with a longitudinal slit and all food contents were carefully collected. Empty stomachs were counted. In stomachs with contents, food items were separated, identified, counted, weighed to the nearest gram and allocated to the following categories: (1) fish (identified to species or, if not possible, counted as “unidentified teleosts”), (2) crayfish (identified to species) and (3) other (i) gammarids, (ii) other macrozoobenthos like *Asellus aquaticus* or *Naididae*, (iii) pupae and larvae of insects, (iv) mussels and snails, (v) water fowl and (vi) angling bait, e.g. boilies or baitfish. Where possible, stomach content was weighed to the nearest gram. Claws of crayfish were counted and divided by two to calculate the number of ingested individuals. The body mass (g) of partially digested prey was obtained from weight–length relationships (Vagnon et al., 2022). The diet of catfish was characterized in terms of prevalence (percentage of stomachs containing each species), richness (number of prey species consumed), prey number consumed, biomass (prey weight) and relative biomass (total prey weight in grams divided by individual wet weight of catfish in kilograms).

To compare the feeding patterns of catfish from lakes and rivers, we first calculated the species diversity index *H* for each site according to the following formula:

(6) $$H=-\sum \left[\left(p_{i}\right)\;\ln\left(p_{i}\right)\right]$$

where *p*_*i*_ is the proportion of individuals of each prey species belonging to the *i*th species of total number of consumed individuals. To express how evenly the prey was distributed, we further calculated evenness *E* for each sampling site as follows:

(7) $$E=\frac{H}{\ln\left(k\right)}$$

where *k* is the number of different prey found in the stomachs of all catfish taken from one sampling site.

All statistics were run on JMP Pro 17.2.0 (64 bit, SAS Institute).

## 3 RESULTS

### 3.1 Growth

The TL at age 10 differs between sampling sites (Table 2), with a striking 57% difference (49.2 cm; Table 2) between River Lower Neckar (135.8 cm) and Lake Karsee (86.5 cm). The general linear model for length at age 10 (*n* = 22, *r*_adj_^2^ = 0.72, *p* < 0.0001) revealed a significant impact of water body size (*F*_2,2_ = 10.31, *p* < 0.01) and presence of gobiids (*F*_1,1_ = 5.69, *p* < 0.05) on the model outcome. No significant associations were found with mean number of summer days (*F*_1,1_ = 2.26, *p* > 0.05), the presence of non‐native crayfish (*F*_1,1_ = 2.04, *p* > 0.05) or waterbody type (river/lake) (*F*_1,1_ = 0.01, *p* > 0.05) (Table 2).

### 3.2 Growth at age 2 and 3

The mean increase in TL in the second year of life was 14.4 ± 5.4 cm (mean ± SD). The general linear model for growth during the second year (*n* = 194, *r*_adj_^2^ = 0.28, *p* < 0.05) revealed a significant influence of temperature (*F*_1,1_ = 7.37, *p* < 0.05), while catchment (*F*_3,3_ = 1.86, *p* > 0.05) and site type (*F*_1,1_ = 0.01, *p* > 0.05) had no effect. For the third year of life, the mean increase in length was 15.3 ± 6.9 cm (mean ± SD). As for year 2, the general linear model for growth in the third year (GLM, *n* = 152, *r*_adj_^2^ = 0.18, *p* < 0.05) also revealed a significant influence of temperature (*F*_1,1_ = 3.91, *p* < 0.05), but no effect for either catchment (*F*_3,3_ = 0.58, *p* > 0.05) or site type (*F*_1,1_ = 0.13, *p* > 0.05).

### 3.3 Growth during different time periods

**FIGURE 3:**
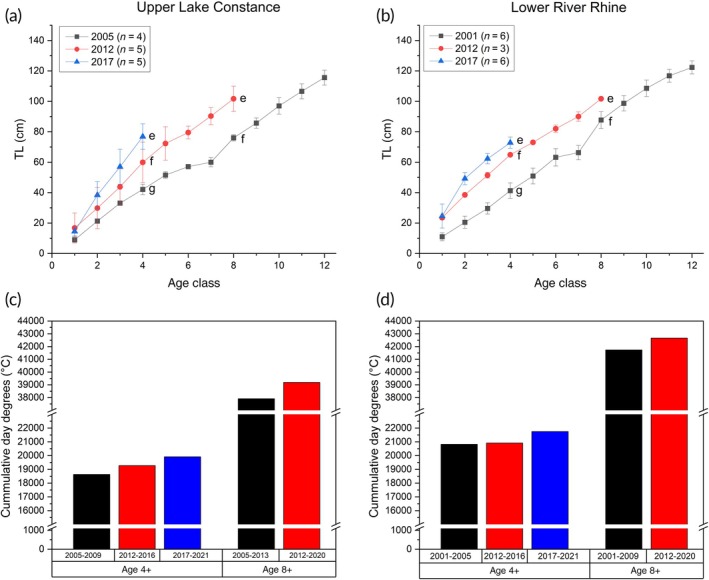
Growth in total body length (cm) for catfish hatched in different years in (a) Upper Lake Constance and (b) in Lower River Rhine. Different letters at age class 4 or 8 indicate significant differences (Tukey's HSD test, *p* < 0.05). The cumulative day degrees derived from air temperature near (c) Upper Lake Constance and (d) Lower River Rhine are given as a proxies for heat budgets available to 4‐ (4+) and 8‐(8+)year‐old catfish *Silurus glanis* growing up at different times. TL, total length.

Sampling from the sites in Upper Lake Constance and Lower River Rhine was sufficient to reconstruct growth trends for catfish hatched at different times (Figure 3). At both sites, catfish hatched in 2017 were significantly longer at age 4 than those hatched in 2012 or 2005 (Lake Constance) or 2001 (River Rhine) (*t*‐test, *p* < 0.05; Figure 3). Furthermore, in both sites, catfish hatched in 2012 achieved significantly greater lengths at ages 4 and 8 than those hatched in earlier years (Tukey's HSD test, *p* < 0.05; Figure 3). These observations correspond to increasing temperature over time. At both sites, the cumulative heat budget for 4‐year‐old catfish was higher for catfish hatched in 2017 (Figure 3) than those that had hatched earlier. Catfish hatched in 2012 were also exposed to higher temperatures as 4‐ or 8‐year‐olds than fish hatched in 2005 (Upper Lake Constance) or 2001 (Lower River Rhine) (Figure 3).

### 3.4 Abundance

**FIGURE 4:**
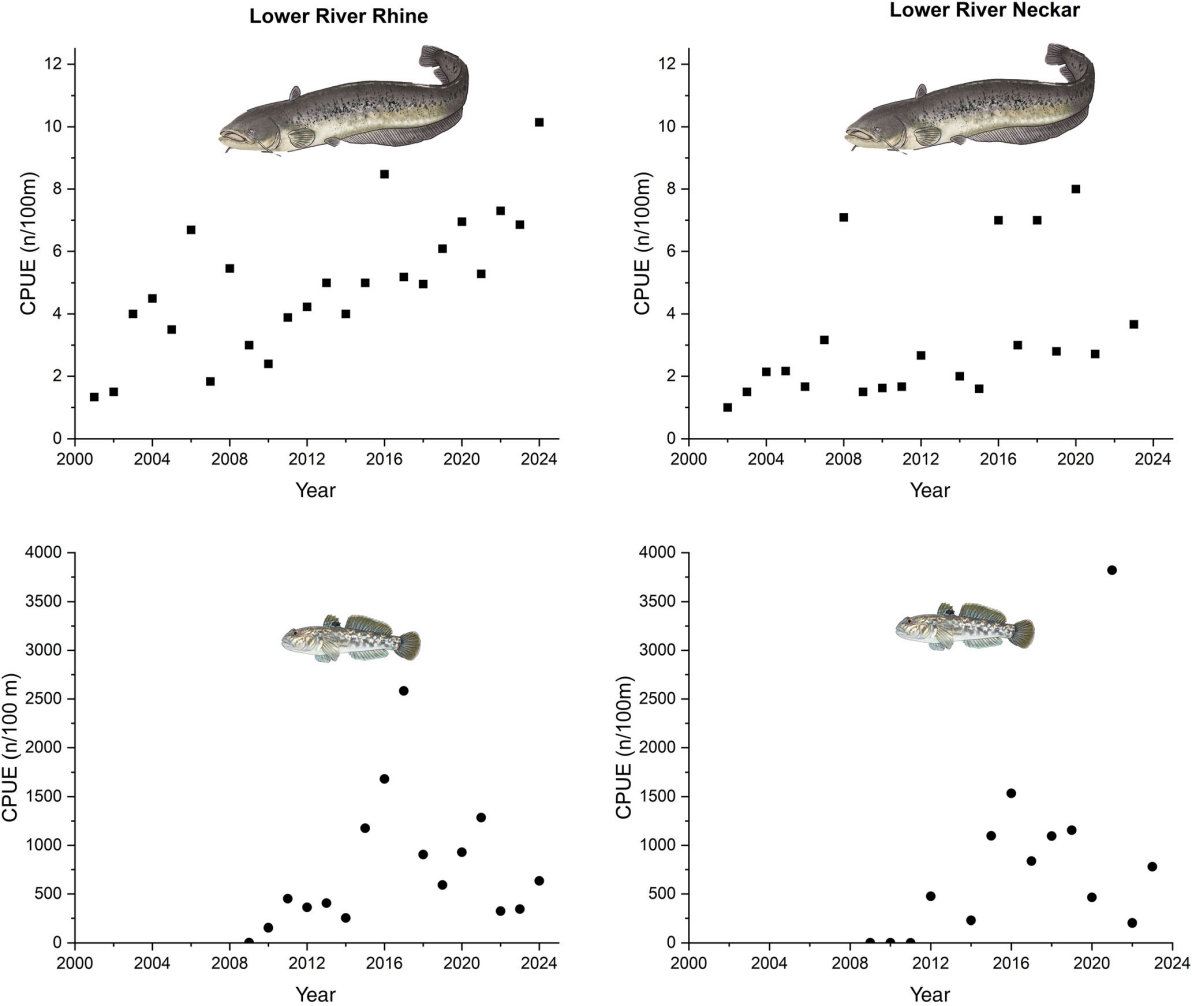
The catch per unit effort (CPUE) of catfish (above) and gobiids (below) in the Lower River Rhine (left) and in the Lower River Neckar (right) in the period from 2002 to 2023.

In the Lower River Rhine and the Lowe Neckar the CPUE of catfish increased continuously (Figure 4) and gobiids were first detected in 2009 (Figure 4). The GLM (*n* = 23, *r*_adj_^2^ = 0.29, *p* < 0.05) revealed a positive significant influence of gobiid abundance on the catfish CPUE (*F*_1,1_ = 6.73, *p* < 0.05), but not air temperature (*F*_1,1_ = 2.21, *p* > 0.05) or the interaction of air temperature and gobiids (*F*_1,1_ = 0.11, *p* > 0.05). Similarly in the Lower Neckar, the GLM (*n* = 22, *r*_adj_^2^ = 0.33, *p* < 0.05) revealed a positive significant impact of gobiid abundance on the catfish CPUE (*F*_1,1_ = 8.31, *p* < 0.01) and no significant impact of air temperature (*F*_1,1_ = 0.93, *p* > 0.05). However here, the interaction of air temperature and gobiids did exert a significant effect (*F*_1,1_ = 4.85, *p* < 0.05).

### 3.5 Diet

Of 573 analysed stomachs, 303 (52.9%) contained prey. Empty stomachs were found in all sampling months in both lakes and rivers (Figure S1). In rivers, highest prey prevalence was found during April, July, September and October (60%–87%; Figure S1), while in lakes, prevalence peaked in May, June, September, and October (46%–83%; Figure S1).

**FIGURE 5:**
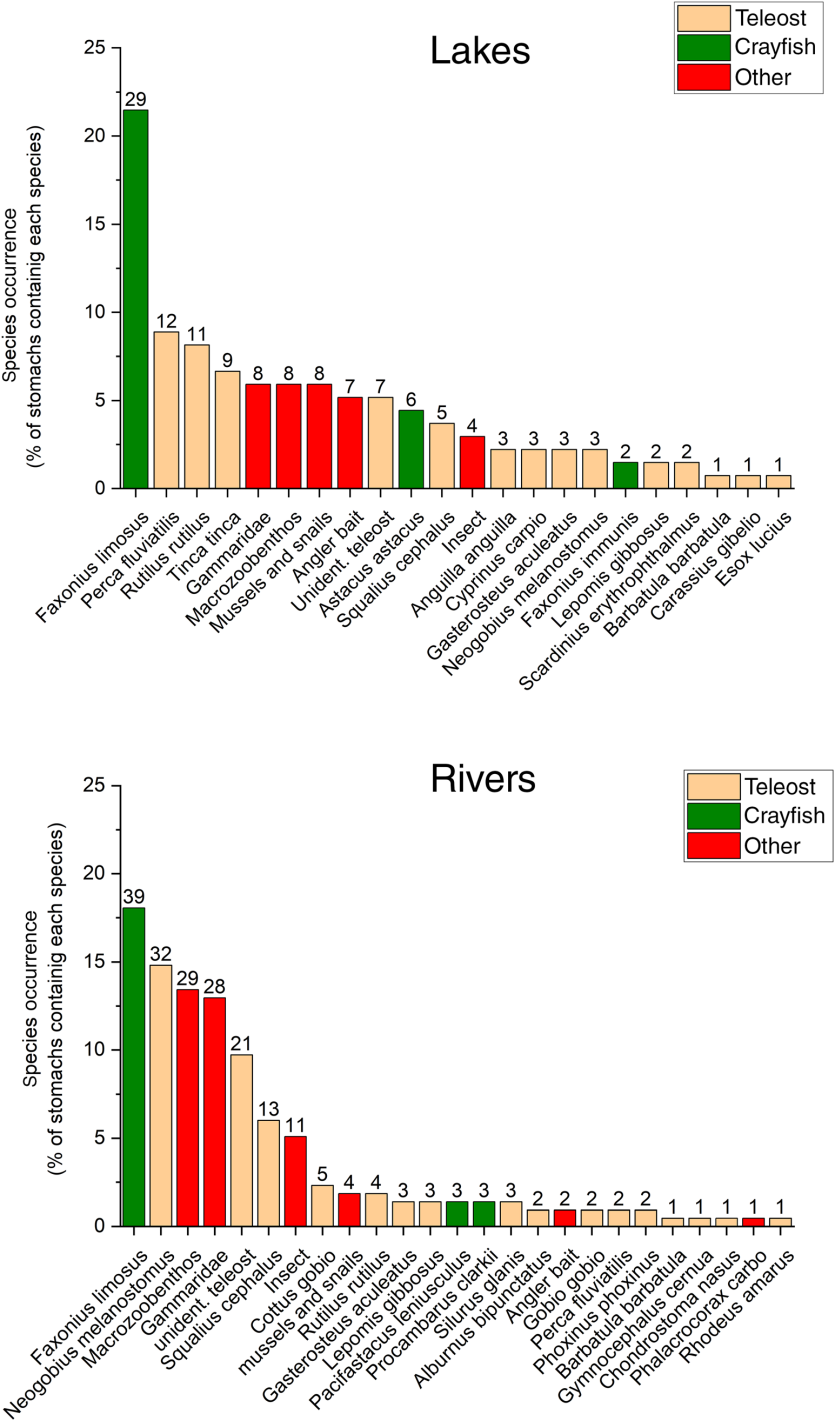
Cumulative occurrence of prey items in catfish (*Silurus glanis*) from different lakes and rivers in southern Germany. Numbers above the columns are absolute counts of stomachs with corresponding prey.

Twenty‐two fish species and five crayfish species were identified as prey items (Figure 5). The most common prey was the non‐native spiny‐cheek crayfish (*Faxonius limosus*), occurring in 21.5% of all analysed stomachs from lake‐caught catfish and in 18.1% of catfish sampled from rivers (Figure 5). In total, spiny‐cheek crayfish were found in catfish from 67% of sampled sites (16 out of 24) and catfish of nearly all sizes, from 13 cm up to 210 cm (mean TL 74 cm ± 37 cm SD) foraged on crayfish. The next most frequent prey in lake catfish were the ubiquitous native perch (*Perca fluviatilis*, 8.8%), roach [*Rutilus rutilus* (L.), 8.1%] and tench [*Tinca tinca* (L.), 6.6%]. Average length of these fish prey was around 9 cm TL (perch mean TL 8.7 cm ± 2.4 cm SD, roach mean TL 8.6 cm ± 3.5 cm SD, tench mean TL 8.5 cm ± 6.7 cm SD). In catfish from rivers, the invasive round goby (*Neogobius melanostomus*, 14.8%) and native chub [*Squalius cephalus* (L.); 5.8%] were the most abundant fish prey items (Figure 5). Round gobies (mean TL 6.8 cm ± 2.8 cm SD) were found in stomachs in the Rivers Rhine, Neckar and Kinzig and in one lake (a gravel pit next to the River Neckar, in the Max‐Eyth‐See), but were absent from all samples from the Danube catchment and Lake Constance and its tributaries (Table S1). Round goby were consumed by catfish of all length classes (15–201 cm TL, mean TL 93 cm ± 40 cm SD). *Gammaridae* were another important food source in both lakes and rivers (Figure 5). Rare and threatened endemic nase [*Chondrostoma nasus* (L.)] and bitterling [*Rhodeus amarus* (Bloch 1782)] each were found in single specimens of catfish and spirlin [*Alburnoides bipunctatus* (Bloch 1782)] only in two catfish (Figure 5) while other endemic, small‐bodied but less rare fish species like bullhead (*Cottus gobio* L.) or minnow [*Phoxinus phoxinus* (L.)] were found in stomachs from catfish sampled from just two rivers (the Kinzig and the Jagst). In both rivers, several of those small‐bodied species formed the bulk of the diet of sampled catfish and in most cases they were the only prey items found. Prey longer than 20 cm TL, like eel [*Anguilla anguilla* (L.)], pike (*Esox Lucius* L.) and carp (*Cyprinus carpio* L.) were only found in individual catfish (Figure 5). Examples of cannibalism were rare, with small catfish (<40 cm TL) only found occasionally in the stomachs of large individuals in just three rivers (Table S1). The highly endangered endemic noble crayfish [*Astacus astacus* (L.)] were found exclusively in samples from Lake Argensee (Table S1), but appeared in six out of 10 catfish from that location. The non‐native crayfish *Faxonius imunis*, *Pacifastacus leniusculus* and *Procambarus clarkii* were recorded as single individuals in single sites (Table S1). No mammals were found and a single cormorant [*Phalacrocorax carbo* (L.)] in a catfish from the River Kinzig (TL 206 cm) was the only bird prey recorded. Angling baits, such as boilies or sardines wrapped in a rubber band (to hold on the hook), were found in nine catfish from three sites (Table S1).

**FIGURE 6:**
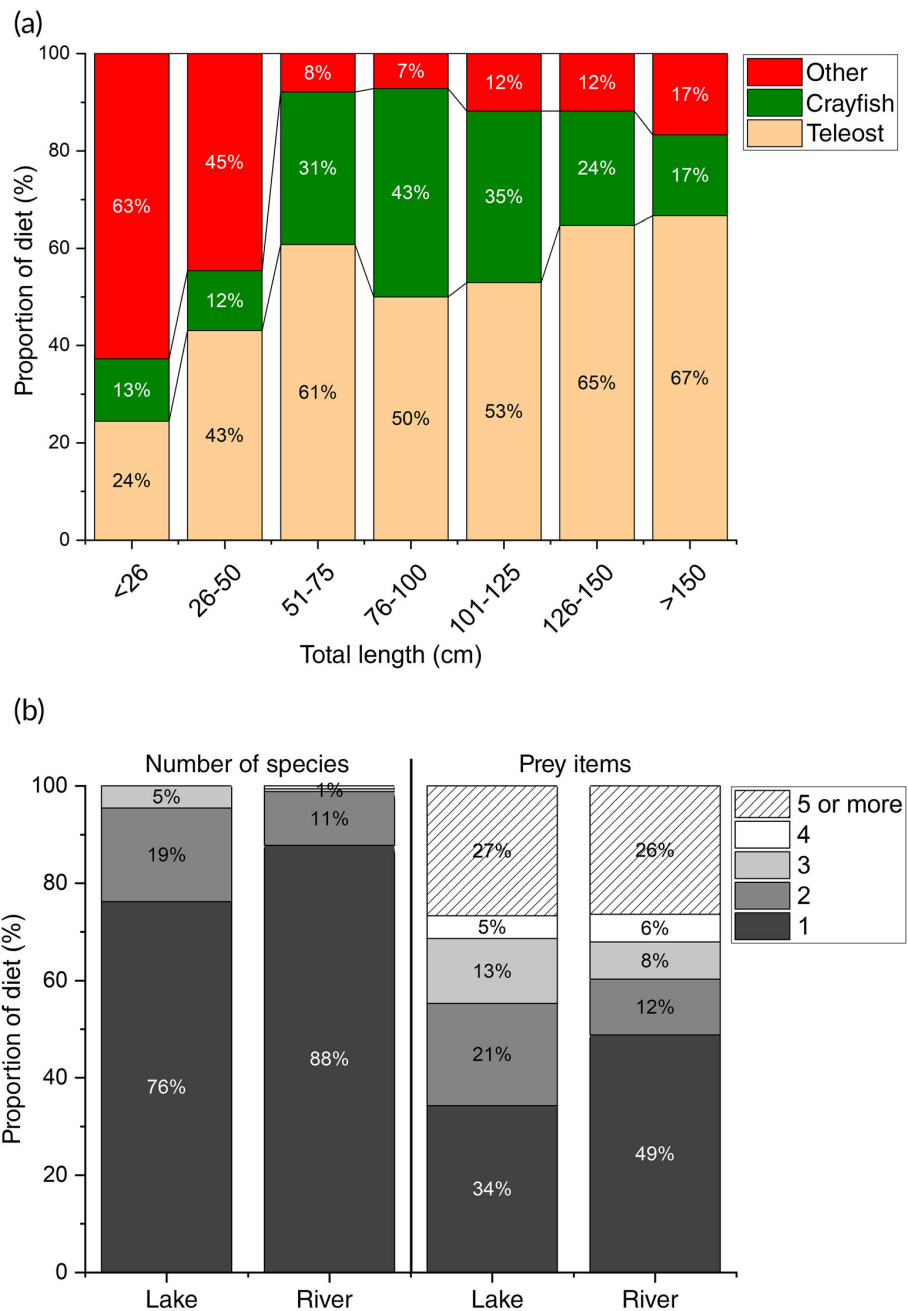
(a) The importance of different prey classes as a proportion in numbers in diet in relation to length classes and (b) the proportion of prey richness (number consumed different taxa, left) and consumed individuals (absolute numbers, right) found in the stomachs of *Silurus glanis* in different lakes and rivers in southern Germany.

The general prey richness per catfish was low, with more than 75% of analysed stomachs yielding one single taxon (Figure 6). Two different taxa were found in 19% of stomachs from catfish from lakes and in 11% in catfish from rivers (Figure 6). Three or more taxa were found very rarely (Figure 6). No difference in prey richness was found between lakes and rivers (Pearson, chi^2^ = 7.0, *p* > 0.05). Furthermore, the numbers of prey consumed were low, with most stomachs containing one or, less often, two individuals of a certain prey type (Figure 6). Only a quarter of analysed stomachs from both lake and river sites contained five or more prey items (Figure 6). There was difference in the number of consumed prey items by lake and river catfish (Pearson, chi^2^ = 10.0, *p* > 0.05).

The importance of prey varied with catfish size: individuals of less than 50 cm TL consumed high quantities of food items such as gammarids, insects, snails or mussels (Figure 6). The importance of fish increased with body length, while crayfish were generally found to be an important food source for catfish of all length classes, but especially for individuals larger than 50 cm TL (Figure 6).

The diversity of prey in the stomachs was not statistically different between site type (*t*‐test, *p* > 0.05), with indices of 0.93 ± 0.31 (mean ± SD) for lakes and 0.80 ± 0.18 (mean ± SD) for rivers. The prey taxa were more unevenly distributed in the stomachs of river catfish than lake specimens (*t*‐test, *p* > 0.05), with index of evenness (*E*) significantly higher for lakes (0.68 ± 0.17, mean ± SD) than for rivers (0.47 ± 0.15, mean ± SD). The River Middle Neckar proved to be a significant outlier, with an *E* value three times the standard deviation, and was excluded from the data set. The range of both indices is given in the (Table S1).

Regarding the relative consumed biomass, no significant difference (Kruskal‐Wallis test, chi^2^ = 0.01, *df* = 1, *p* > 0.05) was apparent between catfish from lakes (35.5 ± 53.1 g kg^−1^, mean ± standard deviation SD) and rivers (40.0 ± 57.8 g kg^−1^, mean ± SD). However, the relative consumed biomass followed a negative logarithmic regression (*y* = −45.95ln[*x*] + 212,74, *r* ^2^ = 0.45), decreasing with increasing catfish length. Catfish less than 30 cm consumed up to four times more biomass in relation to body weight compared to individuals of more than 150 cm (Figure S2).

## 4 DISCUSSION

The findings of the present study support our hypothesis of changing environmental conditions influencing the carrying capacity for catfish in its native home range resulting in the species' increasing abundance and growth. Changes in both prey supply (bottom‐up) (Haugen et al., 2007; Kennedy et al., 2018) and habitat availability (Ayllón et al., 2012; Minns et al., 1996) likely explain why the populations of this apex predator continues to grow. Firstly, ongoing climate change has raised water temperature, boosting reproduction, recruitment and growth (Copp et al., 2009). Secondly, the arrival of a novel and abundant invasive gobies has resulted in almost unlimited food, eliminating bottom‐up control of the catfish population.

This outcome is relevant for many different water bodies, where temperatures are set to increase further in the future (van Vliet et al., 2013). The results strongly suggest that ongoing warming may expand the availability of potential habitat within catfish native range into previously cool upstream river stretches, but further studies are required to validate this assumption. Functionally, given sufficient food, temperature will elevate metabolic rate, and thereby enhance growth and reproduction of catfish in rivers and reservoirs where they currently exist (Britton et al., 2007; David, 2006). The dietary plasticity of *S. glanis* evidenced in our study, where 22 different fish species, five crayfish species and many different macrozoobenthos taxa were consumed, will help to meet increasing demand for food for increasing populations and will most likely provide a competitive advantage over obligatory piscivorous fish such as pike (*Esox lucius*) with their smaller isotopic niche (Vejřík et al., 2023), eventually resulting in a shift of overall dominance to catfish. As effective generalists, catfish are well placed to adapt to novel prey resources (Copp et al., 2009; Vejřík et al., 2023; Vejřík, Vejříková, Blabolil, et al., 2017) and it is therefore not surprising that invasive gobiids (Baer, Hartmann, & Brinker, 2017; Gaye‐Siessegger et al., 2022) and crayfish (Chucholl, 2016) have become major contributors to the diet of catfish. The non‐native round goby has proved a particularly successful invasive species in central Europe, increasing strongly in abundance due to its plasticity, high reproduction rate and adaptability to anthropogenically modified habitats (Brandner et al., 2013, 2015; Cerwenka et al., 2023). However, both gobiids and crayfish are smaller prey than the large gape of catfish suggests, but larger prey were rarely observed in our study. Even if we did not have detailed information about the overall prey availability, data from the electric fishing surveys in all study site (Table S1) showed that larger alternative prey were available, while our data documents almost generally prey sized around 10 cm. This is in line with observations in native (Wysujack & Mehner, 2005) and non‐native populations (Vagnon, Bazin, et al., 2022). The importance of crayfish as a food source is also supported by other studies (Vagnon, Cattanéo, et al., 2022; Vejřík, Vejříková, Blabolil, et al., 2017) and in non‐native catfish populations, invasive and highly abundant crayfish are sometimes the main prey (Carol, 2007; Carol et al., 2009).

Gobies can rapidly become an important component in the aquatic food web of invaded rivers and act as an important prey for native catfish, which has been shown previously in one European mid‐sized lowland river (Mikl et al., 2017). Catfish prefer feeding near to the riverbed and suck benthic prey into their large mouth as they get close (Copp et al., 2009). Gobies are also benthic feeders (Brandner et al., 2013) and when highly abundant are apparently easy to catch for catfish. Furthermore, it is often stated that non‐native prey might be easier targets for predation because they lack effective, co‐evolved anti‐predator defences (Sih et al., 2010). Gobies have been found in the study area since 2009, so an effective co‐evolved anti‐predator strategy against catfish is highly unlikely. Given that goby growth and reproduction is also positively related to temperature (Klarl et al., 2024; Kornis et al., 2012), climate change will most likely boost the density and abundance in warming lowland rivers (Sinclair et al., 2021), resulting in high densities of easy prey for *S. glanis*. In medium and small‐sized rivers and standing water bodies without gobiids, our data showed significantly slower catfish growth rates. In contrast, the occurrence of non‐native crayfish has no significant impact on catfish growth, possibly highlighting the relatively low energy content of crayfish compared to teleosts (Dekar et al., 2010). We assume that the high prevalence of crayfish in catfish diet is because they are widespread and easy to catch, but their low nutritional value compared to gobies or benthic fish (Ruetz III et al., 2009) may limit individual growth. Furthermore, according to our models, the abundance of gobiids has an even stronger positive impact on growth and abundance of catfish than temperature. Similarly, rapid increases in predator abundance and fitness after introduction of a non‐native prey is well documents in terrestrial systems (Sih et al., 2010), therefore based on their high abundance, catchability and nutritional value, gobiids likely are a main driver of the observed expansion of *S. glanis* in its native range.

Overall, the predation pressure on native taxa, including endangered species of fish and crayfish species, will probably increase with increasing catfish distribution and abundance. Indeed, we found catfish stomachs containing large numbers of individuals of endangered species, such as bullhead in the River Jagst and noble crayfish in Lake Argensee. Significant selective predation of endangered species by catfish was previously shown in a study in southwestern France, where in a fishway of the River Gironne, 35% of migrating adult salmon *Salmo salar* L. were predated by *S. glanis* (Boulêtreau et al., 2018). These species are relatively easy prey, especially where they concentrate around man‐made installations like weirs and turbines seeking routes for up‐ and downstream migration. Catfish appear extraordinarily capable of adapting to such opportunities (van Rijssel et al., 2024).

To protect endemic communities, especially endangered and migratory species, and to reduce predation pressure linked to increasing catfish stock, new management concepts are urgently required. This holds especially true in hotspots for species of high conservation priority or areas where catfish impacts are particularly severe. Measures such as rigorous removal of catfish by angling in those areas (e.g. near fish ladders, weirs and hydropower plants) could be implemented quickly and effectively. Furthermore, the increasing range and abundance of catfish, even within the natural distribution, suggest that size or bag limits for anglers are not required. Indeed, the removal of small individuals appears to be at least as important as the removal of larger animals, as our data showed that catfish of less than 30 cm consume approximately four times more biomass per kilogram of body weight than individuals exceeding 1.5 m. However, such measures are diametrically opposed to the preference of anglers for large fish and catch and release practices (Fromherz et al., 2024). Appropriate fisheries management will thus even become more important in the coming years to decrease the threat to native species and secure ecosystem integrity (De Santis et al., 2024). Follow‐up studies are also needed to assess the likely extent of catfish range expansion and increased predation pressure. Such studies could be used to identify key points where increased removal of catfish is most urgent and also to identify river areas where catfish intrusion must be avoided at all costs (e.g. spawning areas of particularly endangered species). Our results suggest that these studies will require updated growth functions to account for dynamic increases in growth with climate warming and the spread of invasive species to avoid underestimating growth‐linked consequences for predation or propagation potential.

Regarding feeding preferences, three further points should also be considered in the future. First, the rare instances of cannibalistic behaviour recorded in our study concur with the observations of other authors (Ferreira et al., 2019; Haubrock et al., 2020; Vagnon, Bazin, et al., 2022; Vejřík, Vejříková, Blabolil, et al., 2017) and may hinder self‐regulation, which is normally a common stabilizing mechanism for other aquatic apex predators (Haugen et al., 2007). Secondly, our results showed that diet size structure and composition changes only slightly with age. The only exceptions, as found elsewhere, is that very young and small catfish <25 cm total length prefer small benthic organisms (Copp et al., 2009; Haubrock et al., 2020). Above this size, the amount of fish in the diet of catfish increases (Orlova & Popova, 1987). Third, the tendency of catfish stomachs to contain single prey taxa may point to individual prey‐type specialization, at least at specific periods of time. We assume that despite their overall dietary plasticity, catfish tend to specialize on a particular food source when it is easily available, for example when certain fish or crustacean species gather in large numbers for reproduction. However, as this kind of food source tends to be available for limited periods of time, other sources are likely to become important during the rest of the year, leading to further temporal specialization and broad overall diet extending beyond aquatic species. Similar results elsewhere have been reinforced with integrative stable isotope analyses (Vagnon, Bazin, et al., 2022; Vejřík et al., 2023), and a study in Czech lakes using repeated dietary analyses matches our empirical evidence of short‐term specialization within long‐term generalism in *S. glanis* populations (Vejřík, Vejříková, Blabolil, et al., 2017).

## 5 CONCLUSIONS

The baselines determining the carrying capacity of the catfish *S. glanis* within the species' native range seem to have shifted, explaining why abundance and growth have increased over recent years or even decades in three large catchments (Lake Constance, River Rhine and River Neckar). This development is most likely driven by increasing water temperatures and the spread and density increase of non‐native gobiids, which catfish are well placed to exploit. The impact of increasing predation pressure on the natural fish fauna is mitigated to a certain degree by the presence of non‐native species such as invasive crayfish and gobiids, which can make up large proportions of the catfish diet, but at the same time the spread of such species is facilitating the range expansion in catfish. Ongoing climate change will likely exacerbate this effect. The findings of our study suggest that it is highly likely that native *S. glanis* will continue to spread and further increase in abundance as the limits related to carrying capacity continue to shift. Consequently, the conservation of native species in prioritized areas requires a change in catfish management with no limit on size and bag limits, and a strict enforcement of catch‐and‐release bans in such areas.

## AUTHOR CONTRIBUTIONS

Jan Baer: Formal analysis, data curation, investigation, sampling, validation, visualization, writing – original draft, writing – review and editing. Stephan Hüsgen: Sampling, visualization, writing – review and editing. Matthias Fromherz: Formal analysis, data curation, sampling, investigation. Juergen Geist: Validation, visualization, writing – original draft, writing – review and editing. Alexander Brinker: Project administration, resources, software, supervision, investigation, validation, visualization, writing – original draft, writing – review and editing.

## Supporting information


**DATA S1.** Supporting Information.
