# Supplementary material for: Drivers of the range expansion of the European catfish (Silurus glanis) within its native distribution
Source: J Fish Biol. 2025 Jun 12;107(3):1030–44. doi: 10.1111/jfb.70099 (PMC12463768; doi:10.1111/jfb.70099)
Supplement: Supplementary file 1 — DATA S1. Supporting Information. [file JFB-107-1030-s001.docx]

**Drivers of the range expansion of the European catfish (*Silurus glanis*) within its native distribution**

**Supplement**

**Figure 1: Proportion of stomachs with (consumed) or without (empty) prey during different month for all sampled catfish (above) and separated for catfish from lakes (middle) and from rivers (below). Numbers above columns representing the sampling size per month.**

**Table 1: List of consumed fish species, crayfish species and other consumed prey of catfish sampled, the Shannon-Wiener and Evenness Index, the altitude, the number of samplings and the sampling months in the 12 rivers (number in brackets from 1-12) and in the 12 lakes (numbers in brackets from 13-24)**


**Figure 2: The relative consumed biomass in relation to body length (TL in cm) for all sampled catfish (with prey in the stomach); the relative consumed biomass followed a negative logarithmic regression (red dotted line, function and r² is given).**
